# Downregulation of L-type amino acid transporter 1 expression inhibits the growth, migration and invasion of gastric cancer cells

**DOI:** 10.3892/ol.2013.1342

**Published:** 2013-05-08

**Authors:** LIANGHUI SHI, WENPING LUO, WENBING HUANG, SHISUI HUANG, GUANGYAN HUANG

**Affiliations:** 1 Department of Surgery, The First Affiliated Hospital of Wannan Medical College, Wuhu, Anhui 241001;; 2 Chongqing Key Laboratory for Oral Diseases and Biomedical Sciences, Chongqing Medical University, Chongqing 400117;; 3 The Affiliated Hospital of Stomatology, Chongqing Medical University, Chongqing 400117;; 4 Department of Pathology, The First Affiliated Hospital of Nanjing Medical University, Nanjing, Jiangsu 210029, P.R. China

**Keywords:** L-type amino acid transporter 1, gastric cancer, proliferation, migration, invasion

## Abstract

Gastric cancer is the second leading cause of cancer-related mortality worldwide. Identifying the molecules that play critical roles in the development of gastric cancer, and clarifying their mechanisms, will contribute to the development of novel molecularly targeted therapeutic drugs. Recently, the large (L)-type amino acid transporter 1 (LAT1), a glycoprotein that transports amino acids through the cell membrane when associated with CD98hc, has been demonstrated to be overexpressed in various types of cancer, and to regulate multiple biological process, including cell growth, migration and invasion. However, the involvement of LAT1 in gastric cancer remains unclear. In the present study, stable gastric cancer cell lines with a LAT1 knockdown were established by transfection of constructs with inserted short (sh) RNAs, in order to clarify the role of LAT1 in gastric caner. A significant decrease in LAT1 expression was observed in the established LAT1-silenced SGC7901 cells compared with the corresponding control cells; however, the expression levels of its partner, CD98hc, were not altered. Furthermore, downregulation of LAT1 expression inhibited the proliferation, migration and invasion of gastric cancer cells. In addition, the decreased expression of LAT1 induced cell cycle arrest in the G_1_/M phase. These findings suggested that LAT1 may be significant in the progression and metastasis of gastric cancer, and may be developed as a therapeutic target for cancer therapy.

## Introduction

Gastric cancer remains the second leading cause of cancer-related mortality worldwide. With the development of novel diagnostic markers and effective treatments, the morbidity and mortality rates of this disease have significantly decreased worldwide, particularly in Asian countries. Although an increasing number of molecules that play critical roles in the development of gastric cancer have been identified, the pathophysiological progression of the carcinogenesis is far from clear, and the relative five-year survival rate of gastric cancer patients remains low (1). In recent years, accumulating studies have focused on the contribution of the metabolism of cancer cells in carcinogenesis.

Tumor cells require steady and sufficient nutrition to maintain their energy supply and the protein synthesis required for rapid growth. Amino acid transporters are commonly upregulated in cancer cells for their supply of amino acids. Large (L)-type amino acid transporter 1 (LAT1) is encoded by the *SLC7A5* gene and belongs to system L, which is an Na^+^-independent system. LAT1 mainly transports large neutral, branched and aromatic amino acids, including leucine, isoleucine and tyrosine, the majority of which are essential amino acids (2). LAT1 therefore has a significant role in cell metabolism (3). LAT1 has been demonstrated to be upregulated in proliferative tissue, cancer cell lines and numerous types of human cancer tissue, including lung, colon, breast, prostate, head and neck, and ovarian cancer, as well as in gliomas (2–5). In non-small cell lung carcinoma (NSCLC), the increased expression of LAT1 is not only correlated with histological type, disease stage and metastasis, but also with the five-year survival rate (6). In gliomas, the overexpression of LAT1 is correlated with pathological grade, proliferation and angiogenesis (7). Recently, Ichinoe *et al* revealed that LAT1 was overexpressed in gastric cancer, suggesting that it may be involved in the oncogenesis of gastric cancer (8).

LAT1 has been demonstrated to promote cell proliferation, migration and invasion in certain cancer cell lines, including gliomas and ovarian and oral cancer (7). This protein is involved in cancer progression and metastasis, and functions by forming a heterodimeric complex with another glycoprotein, CD98hc. The heavy chain, CD98hc, recruits the light chain, LAT1, in the plasma membrane through covalent association (9). LAT1 may be upregulated or activated by the PI3K/Akt, mTOR, MAPK and c-myc signaling pathways. This upregulation results in an increase of amino acids transported to the plasma, and the subsequent activation of the mTOR signaling pathway, which is important in protein synthesis and supplying energy (9). CD98hc has been demonstrated to link to intergrin β in order to regulate the intergrin signaling pathway that is involved in cell proliferation, survival, migration and epithelial adhesion/polarity (9). The role of LAT1 and its signaling pathway in gastric cancer are currently unclear.

In the present study, two plasmids were constructed with different short (sh)RNAs inserts that targeted LAT1, which resulted in a LAT1 knockdown. A corresponding control shRNA plasmid was also constructed. Subsequently, stable SGC7901 cell lines with a LAT1 knockdown, and the corresponding control cell lines, were established by transfection with these plasmids. The efficiency of LAT1 silencing and the expression levels of CD98hc were then confirmed. The effects of silencing the LAT1 expression on the proliferation, cell cycle, migration and invasion of these SGC7901 cells was then further investigated. These results suggested that the down-regulation of LAT1 expression using shRNAs inhibited the proliferation, migration and invasion of gastric cancer cells. These findings suggested that LAT1 is important in gastric cancer, and that it may be developed as a therapeutic target.

## Materials and methods

### Reagents

Lipofectamine 2000 transfection reagent was purchased from Invitrogen Life Technologies (Carlsbad, CA, USA). The LAT1 antibody was purchased from Beijing Zhongshan Golden Bridge Biotechnology Co., Ltd., (Beijing, China) and the CD98hc antibody was purchased from Santa Cruz Biotechnology, Inc. (Santa Cruz, CA, USA). The actin antibody was purchased from Bioworld Technology, Inc. (St. Louis Park, MN, USA).

### Construction of plasmids

Two sets of shRNAs targeting *SLC7A5* (GenBank, NM_003486), which encodes LAT1, were designed according to the principles of shRNA design. The oligonucleotide sequences of these two shRNAs were as follows: 5′-GGGAACATTGTGCTGGCATT-3′, targeting at 793 bp; and 5′-GCATTATACAGCGGCCTCT-3′, targeting at 808 bp. The sequence of the non-targeting shRNA was 5′-GTTCTCCGAACGTGTCACGT-3′; this served as the control. The loop and stop sequences used were TTCAAGAGA and T6, respectively. The digestion site of *Pst*I (sequence CACC) was added to the 5′ end of the sense strands, and the digestion site of *Bam*HI (sequence GATC) was added to the 5′ end of the antisense strands. The oligonucleotides were synthesized by Shanghai GenePharma Co., Ltd. (Shanghai, China). The sense and antisense strands were annealed and inserted into the pGPU6/GFP/Neo plasmid using T4 DNA ligase. These were transformed in DH5α and selected by kanamycin. The constructs were named LAT1-shRNA1 (targeting at 793 bp) and LAT1-shRNA2 (targeting at 808 bp), confirmed by enzyme digestion and then sequenced by the Invitrogen Corporation Shanghai Representative Office (Shanghai, China).

### Cell lines and cell culture

The human gastric cancer cell line, SGC7901, was obtained from the Shanghai Institutes for Biological Sciences, Chinese Academy of Sciences, China. The SGC7901 cells were divided into four groups and either transfected with the LAT1-shRNA1, LAT1-shRNA2 or LAT1-sh NC constructs, or not transfected, for 48 h. The cells were then selected for two weeks with 400 *μ*g/ml G418. Subsequently, the cell lines were named SGC7901_shRNA1, SGC7901_shRNA2, SGC7901_shNC and SGC7901_blank, respectively. The cells were cultured in RPMI-1640 medium supplemented with 10% fetal bovine serum (Gibco-BRL, Grand Island, NY, USA) at 37°C in a humidified atmosphere consisting of 5% CO_2_. The fluorescence of the green fluorescent protein (GFP) encoded by the pGPU6/GFP/Neo plasmids was observed and images were captured by a fluorescence microscope.

### RNA isolation and semi-quantitative RT-PCR

The total RNA was extracted using Trizol reagent (Gibco, Carlsbad, CA, USA) according to the manufacturer’s instructions. The reverse transcription was conducted using M-MLV reverse transcriptase obtained from Promega Corporation (Madison, WI, USA), according to the standard procedure. The PCR reactions were conducted using Taq DNA polymerase (Thermo Fisher Scientific Inc., Rockford, IL, USA) according to the manufacturer’s instructions. The forward (F) and reverse (R) primers used were as follows: LAT1 F, 5′-GCATGCGCAGAGGCC AGTTAA-3′ and R, 5′-TATGGTCAGGAGTCCATCGGG-3′; CD98hc F, 5′-CCAGGTTCGGGACATAGAG-3′ and R, 5′-TGGTAGAGTCGGAGAAGTTGAG-3′; GAPDH F, 5′-AGA AGGCTGGGGCTCATTTG-3′ and R, 5′-AGGGGCCATCCA CAGTCTTC-3′, and were synthesized by Invitrogen Life Technologies (10). The product lengths of LAT1, CD98hc and GAPDH were 537, 326 and 258 bp, respectively. The PCR fragments were separated by 1.5% agarose gel. The quantity of the PCR products of LAT1 or CD98hc were determined by scanning the density of the bands, using Quantity One software (Tanon Science and Technology Co., Shanghai, China) and normalizing to GAPDH.

### Western blot analysis

Cell lysis buffer was purchased from Promega Corporation and stored at 4°C. Protease inhibitors were added immediately prior to use. The cells were harvested and subjected to western blot analysis following the standard procedure. Briefly, 50 mg protein was electrophoresed in 10% sodium dodecyl sulfate-polyacrylamide gel electrophoresis (SDS-PAGE) and transferred to polyvinylidene difluoride (PVDF) membranes. The membranes were blocked with 5% skimmed milk in Tris-buffered saline and Tween-20 (TBST) for 1 h at room temperature. The blots were incubated with an appropriate dilution of the primary antibody for 2 h at room temperature, then rinsed three times with TBST. The rinsed blots were incubated with the secondary antibody for 1 h at room temperature and then rinsed three times with TBST. The signals were visualized with an enhanced chemiluminescence (ECL) detection system (cat. no. RPN2132; Amersham Pharmacia Biotech Inc., Piscataway, NJ, USA). The quantity of the proteins was determined by scanning the density of the bands using Quantity One software and by normalizing to actin.

### Cell proliferation assay

Cell proliferation activity was determined by the 3-(4,5-dimethyl- thiazol-2-yl)-2,5-diphenyltetrazolium bromide (MTT) assay, according to the standard methods. Briefly, cells were seeded in 96-well plates for 1–4 days. Subsequently, 20 *μ*l 0.5% MTT was added to each well and incubated for 4 h at 37°C. The MTT was removed and 150 *μ*l DMSO was added. Absorbance was measured at 490 nm and detected using the Bio-Tek *μ*Quant Universal Microplate Spectrophotometer (Bio-Tek Instruments, Inc., Winooski, VT, USA).

### Cell cycle analysis

The cells were seeded in 6-well plates in triplicate and fixed in 70% ice-cold ethanol for 24 h at 4°C. They were subsequently washed with phosphate-buffered saline (PBS) solution and resuspended in 1 ml staining solution (50 *μ*g/ml propidium iodide and 100 *μ*g/ml RNase A in PBS) for 30 min. The cell cycle distribution was detected by the FC 500 Series Flow Cytometer (Beckman Coulter Inc., Brea, CA, USA) and analyzed by BD CellQuest analysis software (BD, Franklin Lakes, NJ, USA). Each experiment was repeated three times.

### Cell migration assay

Cell migration was measured with the Boyden chamber (Corning Costar Corp., Cambridge, MA, USA). Briefly, 1×10^5^ cells, in 200 *μ*l RPMI-1640 containing 0.1% fetal calf serum, were plated on the upper compartment of the chamber. The conditioned medium, which was obtained from cultured NIH3T3 cells with serum-free medium, was added to the lower chambers. After 24 h, non-migratory cells on the upper surface of the filter were removed completely with a cotton swab. The migrated cells on the lower surface of the filter were fixed with 95% alcohol for 30 min, stained with hematoxylin and eosin and then counted under a microscope. The mean number of migratory cells of the triplicates for each experimental condition was recorded.

### Cell invasion assay

Cell invasion was also measured with the Boyden chamber (Corning Costar Corp.), but the upper side of the filters were coated with 100 *μ*l matrigel (1 mg/ml), which was dissolved in serum-free RPMI-1640 medium. The remaining operations were the same as those of the migration assay. The invaded cells were fixed, stained and counted as described previously.

### Statistical analysis

Unless otherwise stated, all data are presented as the mean ± standard deviation (SD). Statistical significance (P<0.05) was determined by the Student’s t-test or analysis of variance (ANOVA) followed by the assessment of differences using the Statistical Package for the Social Sciences (SPSS) for Windows, Version 16.0 (SPSS, Inc., Chicago, IL, USA).

## Results

### Establishment of stable gastric cancer cell lines with low LAT1 expression levels

**Figure 1.**
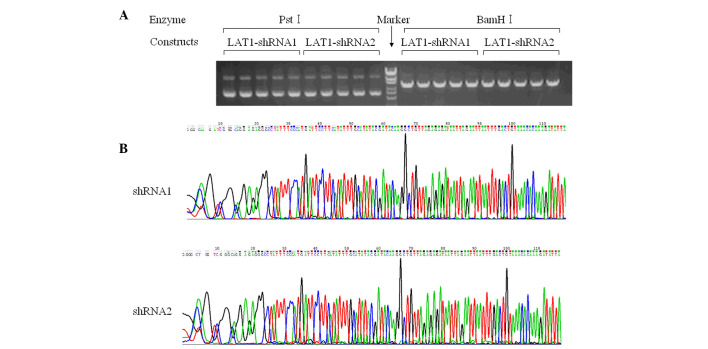
Inserted sequences of large (L)-type amino acid transporter 1 (LAT1) short (sh)RNAs were confirmed by (A) an enzyme digestion method and (B) DNA sequencing. (A) The constructs of pGPU6/GFP/Neo inserted with shRNA1 and shRNA2 were digested with *Pst*I or *Bam*HI, and the DNA fragments were separated by 1.5% agarose gel. (B) The sequences of inserted shRNA1 and shRNA2.

To identify the role of LAT1 in gastric cancer, stable cell lines with a LAT1 knockdown were first established using shRNAs. Two sets of shRNA sequences and one set of control shRNA sequences were designed. Subsequently, the shRNAs were inserted into the pGPU6/GFP/Neo plasmids and named LAT1-shRNA1 (targeting at 793 bp), LAT1-shRNA2 (targeting at 808 bp) and LAT1-shNC (negative control), respectively. Fig. 1A reveals that the enzymes *Pst*I and *Bam*HI digested the plasmids as predicted. Moreover, DNA sequencing confirmed that the sequences were correct (Fig. 1B).

**Figure 2.**
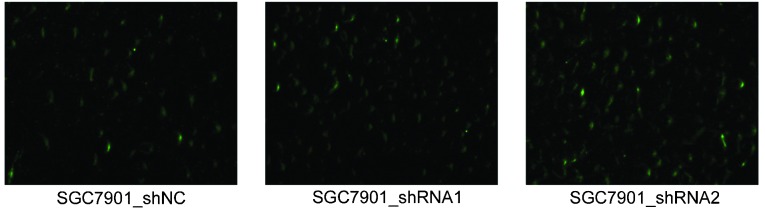
Green fluorescent protein expression in established SGC7901 cells under a fluorescence microscope. Magnification, ×200.

Subsequently, the plasmids were transfected into SGC7901 cells and further selected the positive colonies using 400 *μ*g/ml G418 for two weeks. As demonstrated in Fig. 2, successful expression of the plasmids exhibited green fluorescence under a fluorescence microscope due to the expression of GFP, the coding sequence of which had been inserted into the backbone of the pGPU6/GFP/Neo plasmids. The established cell lines were named SGC7901_shRNA1, SGC7901_shRNA2 and SGC7901_shNC, respectively. SGC7901 cells without transfection were also cultured, and these served as a blank control. These results suggested that the constructs were successfully expressed in the SGC7901 cells.

### Expression levels of LAT1 and CD98hc in the established SGC7901 cells

**Figure 3.**
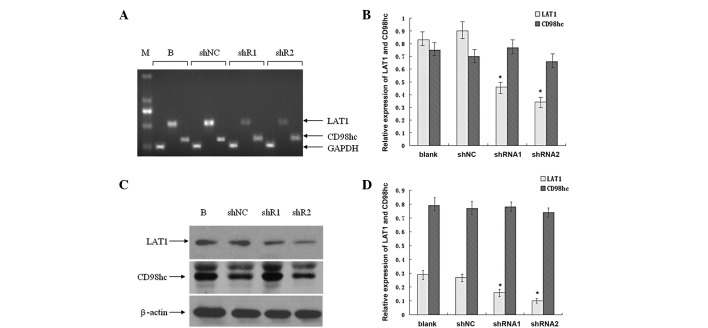
Expression of large (L)-type amino acid transporter 1 (LAT1) and CD98hc in established SGC7901 cells. Total mRNA was extracted from SGC7901_blank, SGC7901_shNC, SGC7901_shRNA1 and SGC7901_shRNA2 cells, and subjected to RT-PCR analysis. (A) RT-PCR products were separated in 1.5% agarose gel. (B) Quantification of the DNA fragments in (A). (C) Whole-cell protein lysates were purified from the four aforementioned cell lines and subjected to western blot analysis. (D) Quantification of the protein bands in (C). Columns, means; bars, SD. *P<0.05 vs. blank. Representatives of three independent experiments. M, marker; B, SGC7901_blank; shNC, SGC7901_shNC; shR1, SGC7901_shRNA1; shR2, SGC7901_shRNA2.

To further determine the expression levels of LAT1 and its functional partner, CD98hc, in the established SGC7901 cell lines, their mRNA levels were detected using RT-PCR analysis. Fig. 3A reveals that the mRNA levels of LAT1 markedly decreased in the SGC7901_shRNA1 and SGC7901_shRNA2 cells compared with the SGC7901_shNC and SGC7901 (blank control) cells, while those of CD98hc only minimally decreased. The density of the mRNA bands was further quantified (Fig. 3B). The decreases in LAT1 in the SGC7901_shRNA1 and SGC7901_shRNA2 cells were significant compared with the SGC7901_shNC cells (P<0.05). Moreover, the knockdown efficiency of shRNA2 (which decreased the LAT1 mRNA expression by 62.1%) was greater than that of shRNA1 (which decreased the LAT1 mRNA expression by 48.9%). In addition, the mRNA levels of CD98hc did not change significantly (P>0.05).

Furthermore, the protein levels of LAT1 were detected by western blot analysis. As demonstrated in Fig. 3C, the LAT1 protein levels were markedly decreased, in the SGC7901_shRNA1 and SGC7901_shRNA2 cells compared with the SGC7901_shNC and SGC7901 cells, but those of CD98hc did not appear to be altered. Additionally, following quantification of the band densities, the present study identified that the LAT1 protein levels were significantly decreased in the SGC7901_shRNA1 and SGC7901_shRNA2 cells compared with the SGC7901_shNC cells (P<0.05; Fig. 3D). Moreover, the knockdown efficiency of shRNA2 was greater than that of shRNA1. Furthermore, the CD98hc levels did not change following quantification and analysis.

These results suggested that the expression of LAT1 was downregulated in the established SGC7901_shRNA1 and SGC7901_shRNA2 cells compared with the SGC7901_shNC and SGC7901 cells, and that the downregulation of LAT1 expression does not affect the expression of its functional partner, CD98hc.

### Downregulation of LAT1 expression inhibits the growth of gastric cancer cells

**Figure 4.**
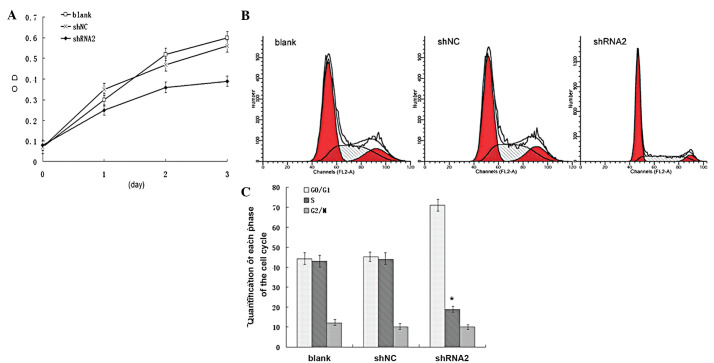
Knockdown of large (L)-type amino acid transporter 1 (LAT1) expression inhibited cell proliferation and induced cell cycle arrest of gastric cancer. (A) SGC7901_blank, SGC7901_shNC and SGC7901_shRNA2 cells were seeded in 96-well plates and subjected to 3-day MTT assay. Points, means; bars, SD. (B) Cell lines were seeded in 6-well plates and subjected to flow cytometry, and analyzed by CellQuest software. (C) Quantification of each phase of the cell cycle. Columns, means; bars, SD. *P<0.05 vs. blank. Blank, SGC7901_blank; shNC, SGC7901_shNC; shRNA2, SGC7901_shRNA2.

To explore the function of LAT1 in gastric cancer, the present study first examined its effect on cell proliferation. Cell proliferation was determined by a 3-day MTT assay in the SGC7901, SGC7901_shNC and SGC7901_shRNA2 cells. Fig. 4A shows that the knockdown of LAT1 expression significantly inhibited the growth of the SGC7901_shRNA2 cells compared with the SGC7901_shNC and SGC7901 cells (P<0.05). Next, the cell cycle of these cells was analyzed by a flow cytometry assay. As shown in Fig. 4B and C, the percentage of SGC7901_shRNA2 cells in G_0_/G_1_ phase was significantly increased (P<0.05), whereas the percentage in the S phase was significantly decreased (P<0.05) compared with the SGC7901_shNC cells. The difference in the percentage of cells in the G_0_/G_1_ and S phases between the SGC7901_shNC and SGC701 cells was not significant (P>0.05). These results suggested that downregulation of LAT1 expression inhibited the growth of SGC7901 cells and induced cell cycle arrest in the G_0_/G_1_ phase.

### Downregulation of LAT1 expression inhibits the migration and invasion of gastric cancer cells

**Figure 5.**
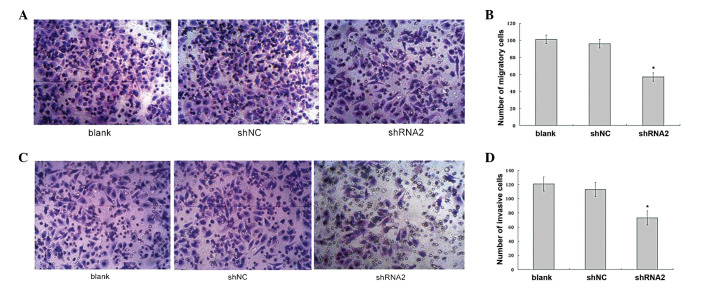
Knockdown of large (L)-type amino acid transporter 1 (LAT1) expression inhibited the migration and invasion of gastric cancer cells. (A) Representative field of migratory cells on the membrane. (B) Average number of migratory cells from triplicate measurements. (C) Representative field of invasive cells on the membrane. (D) Average number of invasive cells from triplicate measurements. Columns, means; bars, SD. *P<0.05 vs. blank. Blank, SGC7901_blank; shNC, SGC7901_shNC; shRNA2, SGC7901_shRNA2.

The role of LAT1 in cell migration was then examined. Fig. 5A shows that the number of SGC7901_shRNA2 cells that migrated to the lower side of the membrane was decreased compared with that of the SGC7901_shNC and SGC7901 cells. The quantitative analysis revealed that the number of migratory SGC7901_shRNA2, SGC7901_shNC and SGC7901 cells was 57±4.7, 96±6.5 and 101±3.4 per field, respectively (Fig. 5B). The difference in the number of migratory cells between the SGC7901_shRNA2 and SGC7901_shNC cells was significant (P<0.05), whereas the difference between the SGC7901_shNC and SGC7901 cells was not significant (P>0.05).

The role of LAT1 in cell invasion was then examined. Fig. 5C shows that the number of SGC7901_shRNA2 cells that invaded to the lower side of the gel and the membrane was decreased compared with that of the SGC7901_shNC and SGC7901 cells. The quantitative analysis revealed that the number of invasive SGC7901_shRNA2, SGC7901_shNC and SGC7901 cells was 73±10.3, 121±11.8 and 113±9.3 per field, respectively (Fig. 5D). The difference in the number of invasive cells between the SGC7901_shRNA2 and SGC7901_shNC cells was significant (P<0.05), whereas the difference between the SGC7901_shNC and SGC7901 cells was not significant (P>0.05).

These results suggested that the downregulation of LAT1 expression inhibits the migration and invasion of gastric cancer cells.

## Discussion

In the present study, the role of LAT1 in gastric cancer was identified by establishing stable cell lines with successful LAT1 silencing and their relative control cell lines. To the best of our knowledge, this study is the first to demonstrate that the downregulation of LAT1 expression inhibits the proliferation, migration and invasion of gastric cancer cells, as well as inducing cell cycle arrest in the G_0_/G_1_ phase.

LAT1, as one of the L-type amino acid transporters, mainly transports large, neutral amino acids, including essential amino acids, and is therefore important in cell metabolism (2). Cell metabolism plays a significant role in cancer progression and metastasis, as rapid growing tumor tissues require a sufficient energy supply and stable protein synthesis (3). Accumulating studies in human tissues utilizing immunohistochemical staining have suggested that LAT1 is upregulated in numerous types of cancer, including gastric carcinoma (8). By detecting the Ki67 label index, VEGF, HIF-1α and numerous other molecules that are significant in cancer progression and metastasis, it has also been demonstrated that the overexpression of LAT1 is correlated with cell proliferation, angiogenesis and hypoxia (11). Moreover, the overexpression of LAT1 is a prognostic marker of lung cancer and gliomas (6,7). To date, studies clarifying the mechanism and signaling pathway of LAT1 *in vitro,* or in the transgenic mouse model, are limited. In the present study, the role of LAT1 was investigated in gastric cancer using a loss-of-function method. A total of two plasmids with LAT1 knockdown, and a control plasmid, medicated by shRNAs were constructed, and then the stable cell lines were established using these constructs through transfection and subsequent selection. Therefore, the results demonstrated the biological features of gastric cancer cells with constitutive LAT1 silencing. These cell lines also provided an *in vitro* model for further clarifying the LAT1 signaling pathway in gastric cancer.

LAT1 links to CD98hc (also known as 4F2hc) by an extracellular disulfide bridge for its cell membrane localization and function. The heavy chain subunit, CD98hc, is a transmembrane glycoprotein that heterodimerizes with one of the light chains, such as LAT1 and LAT2, to form a functional complex for transporting large, neutral amino acids (7). CD98hc is expressed in normal tissue, particularly in the gastrointestinal tract, as well as in tumor tissue. Due to its significance in transporting essential amino acids, genetic disruption of CD98hc results in early embryonic lethality (12). Additionally, overexpression of CD98hc in the gastrointestinal epithelium induces tumorigenesis (9). CD98hc has been demonstrated to regulate cell proliferation, survival, migration and transformation in numerous types of cell lines. Overexpression of CD98hc has been observed in certain types of human cancer tissue, including lung, breast and renal cell cancer (13–15). It has been suggested that LAT1 requires CD98hc for its functional expression, and numerous studies have revealed that the overexpression of LAT1 is correlated with CD98hc expression in human cancer tissue; however, CD98hc expression levels are not always concordant with LAT1 expression levels, suggesting that these two proteins may have separate signaling pathways and functions (7). The present study also found that stably silencing LAT1 did not affect the CD98hc expression levels, but inhibited certain cell biological processes, including growth, migration and invasion. Overall, the findings suggested that LAT1 may have other functions in promoting carcinogenesis, other than through transporting amino acids. Therefore, further studies to clarify the signaling pathway of LAT1 in gastric cancer are required.

It has been documented that the upregulation of LAT1 expression in cancer cells not only induces an increase in the transportation of amino acids, particularly essential amino acids such as leucine, but also activates the mammalian target of rapamycin (mTOR) signaling pathway (5,16). mTOR is a serine/threonine kinase that functions as a complex through interaction with other proteins, including rictor or raptor (17). mTOR complexes respond to growth factors, nutrients and energetic status, and regulate cell growth, survival, autophagy and metabolism (18). mTOR complex 1 (mTORC1) predominantly regulates protein translation through ribosomal p70 S6 kinase (p70S6K) and eukaryotic translation initiation factor 4E-binding protein 1 (4E-BP1). mTORC2 is a kinase that directly activates Akt and other kinases, including protein kinase C (PKC) and serum- and glucocorticoid-induced protein kinase 1 (SGK1) (18,19). The increased transportation of amino acids by the LAT1/CD98hc complex activates the mTORC1 signaling pathway by supplying it with sufficient energy. However, it is possible that other mechanisms exist that are regulated by LAT1 itself; further clarification of the underlying mechanism whereby LAT1 regulates the mTOR signaling pathway is required.

Overall, stable cell lines with successful silencing of LAT1 expression, and relative control cell lines, were established. The downregulation of LAT1 expression was identified to inhibit the proliferation, cell cycle, migration and invasion of gastric cancer cells. These findings suggested that LAT1 is significant in gastric cancer and that it may be developed as a therapeutic target in cancer therapy.
